# Haemodynamic optimisation improves tissue microvascular flow and oxygenation after major surgery: a randomised controlled trial

**DOI:** 10.1186/cc9220

**Published:** 2010-08-10

**Authors:** Shaman Jhanji, Amanda Vivian-Smith, Susana Lucena-Amaro, David Watson, Charles J Hinds, Rupert M Pearse

**Affiliations:** 1 Barts and The London School of Medicine and Dentistry, Queen Mary's University of London, Turner Street, London E1 2AD, UK; 2 Intensive Care Unit, Royal London Hospital, Barts & The London NHS Trust, Whitechapel Road, London E1 1BB, UK

## Abstract

**Introduction:**

Post-operative outcomes may be improved by the use of flow related end-points for intra-venous fluid and/or low dose inotropic therapy. The mechanisms underlying this benefit remain uncertain. The objective of this study was to assess the effects of stroke volume guided intra-venous fluid and low dose dopexamine on tissue microvascular flow and oxygenation and inflammatory markers in patients undergoing major gastrointestinal surgery.

**Methods:**

Randomised, controlled, single blind study of patients admitted to a university hospital critical care unit following major gastrointestinal surgery. For eight hours after surgery, intra-venous fluid therapy was guided by measurements of central venous pressure (CVP group), or stroke volume (SV group). In a third group stroke volume guided fluid therapy was combined with dopexamine (0.5 mcg/kg/min) (SV & DPX group).

**Results:**

135 patients were recruited (*n *= 45 per group). In the SV & DPX group, increased global oxygen delivery was associated with improved sublingual (*P* < 0.05) and cutaneous microvascular flow (*P* < 0.005) (sublingual microscopy and laser Doppler flowmetry). Microvascular flow remained constant in the SV group but deteriorated in the CVP group (*P* < 0.05). Cutaneous tissue oxygen partial pressure (PtO_2_) (Clark electrode) improved only in the SV & DPX group (*P* < 0.001). There were no differences in serum inflammatory markers. There were no differences in overall complication rates between the groups although acute kidney injury was more frequent in the CVP group (CVP group ten patients (22%); pooled SV and SV & DPX groups seven patients (8%); *P* = 0.03) (post hoc analysis).

**Conclusions:**

Stroke volume guided fluid and low dose inotropic therapy was associated with improved global oxygen delivery, microvascular flow and tissue oxygenation but no differences in the inflammatory response to surgery. These observations may explain improved clinical outcomes associated with this treatment in previous trials.

**Trial registration number:**

ISRCTN 94850719

## Introduction

Complications are common following major non-cardiac surgery and represent an important cause of avoidable morbidity and mortality [1-3]. Estimates suggest that as many as 234 million major surgical procedures are performed worldwide each year, around 15% of which fall into a high-risk sub-group [2-4]. With mortality rates of up to 12%, this high-risk surgical population accounts for over 80% of early post-operative deaths [2,3]. Long-term survival is also significantly reduced following surgery, in particular for those patients who develop complications [5-7]. Importantly, survival among patients who develop post-operative complications varies widely between hospitals, confirming both the potential and the need to improve clinical outcomes in this population [8].

The association between low cardiac output, inadequate global oxygen delivery (DO_2_), reduced venous oxygen saturation (mixed venous haemoglobin saturation with oxygen (SvO_2_) central venous haemoglobin saturation with oxygen (ScvO_2_)) and poor outcomes following major surgery is well recognised [9-11]. In several relatively small studies, the use of these variables as treatment end-points for intravenous fluid and inotropic therapy has been associated with improved clinical outcomes [12-18]. It has long been suggested that these beneficial effects relate to improved tissue perfusion and oxygenation. This may prevent the evolution of a tissue 'oxygen debt' and hence reduce the incidence of complications and organ dysfunction [19]. This theory is consistent with the findings of a number of studies demonstrating that impaired tissue microvascular flow and oxygenation are associated with subsequent post-operative complications [20-24]. In patients with severe sepsis, there is some evidence to suggest that abnormalities of microvascular flow may cause tissue hypoxia [25,26], while the use of vasoactive drug therapy has been shown to improve both tissue microvascular flow and oxygenation in this group [27-29]. Importantly, dopexamine, the agent most often used in trials of perioperative cardiac output-guided therapies, has a combination of vasodilator and mild inotropic actions, which may enhance microvascular flow and improve outcomes [12]. The findings of recent systematic reviews suggest that cardiac output guided haemodynamic therapy may have particular beneficial effects on splanchnic perfusion and renal function [30,31]. It is also possible that perioperative haemodynamic optimisation could favourably influence the systemic inflammatory response to tissue injury associated with surgery, thereby reducing the incidence and severity of complications and organ dysfunction.

Clearly, the hypothesis that perioperative cardiac output-guided haemodynamic therapies result in improved tissue microvascular flow and oxygenation is plausible but, after many years, still remains untested. It is also uncertain whether low-dose inotropic therapy offers incremental benefit over the use of fluid alone to achieve cardiac output-related end-points. A detailed understanding of the physiological effects of haemodynamic therapies is therefore necessary to provide a rational basis from which to adapt and refine their use in clinical practice. The aim of this investigation was to evaluate the effects of stroke volume-guided intravenous fluid therapy with and without low-dose dopexamine on tissue microvascular flow and oxygenation and systemic markers of inflammation in patients admitted to critical care following major gastrointestinal surgery.

## Materials and methods

Patients scheduled for admission to critical care following major elective gastrointestinal surgery were eligible for recruitment. Exclusion criteria were refusal of consent, pregnancy, patients receiving palliative treatment only and acute arrhythmias or myocardial ischaemia prior to enrolment. In addition, patients receiving lithium therapy or those with a body mass less than 40 kg were excluded because lithium indicator dilution measurement of cardiac output is not licensed in such patients. The study was approved by the Research Ethics Committee and Medical and Healthcare products Regulatory Agency (UK). Written informed consent was obtained from all patients prior to surgery. Participants were randomly allocated to one of three treatment groups by computer-generated random sequence in blocks of nine. Groups were stratified according to surgical procedure (upper gastrointestinal surgery, lower gastrointestinal surgery and pancreatic surgery involving the gut). Study group allocations were placed in serially numbered opaque envelopes.

### Clinical management

General anaesthesia was standardised and included intravenous fentanyl, propofol and atracurium for induction of anaesthesia and maintenance with inhaled isoflurane in oxygen-enriched air and epidural analgesia. Clinical staff administered intravenous fluids, blood products and, if required, vasoactive drugs in order to maintain routine physiological, haematological and biochemical parameters within normal ranges as follows: pulse rate (60 to 100 bpm), mean arterial pressure (60 to 100 mmHg), central venous pressure (CVP) (6 to 12 mmHg), urine output (> 25 ml/hr), haemoglobin (> 8 g/dl), SpO_2 _(> 94%), temperature (36 to 37°C), serum base excess (-2 to +2 mmol/l) and partial pressure of arterial carbon dioxide (PaCO_2_; 35 to 45 mmHg). Cardiac output monitoring was not used during surgery. Following surgery, all patients were admitted to critical care. For the eight-hour intervention period, either a doctor (SJ) or nurse (AVS, SLA) administered one of three allocated haemodynamic protocols as described below. These protocols are similar to those used in a previous trial [16].

#### CVP group

Intravenous lactated Ringer's solution was administered at 1 ml/kg/hr for maintenance requirements. Patients received additional 250 ml fluid challenges with intravenous colloid solution (Gelofusine, BBraun, Melsungen, Germany) to achieve an optimal value of CVP. Colloid solution was administered in one or more rapid boluses to achieve a sustained rise in CVP of at least 2 mmHg for 20 minutes or more. If CVP decreased, fluid challenges were repeated to establish whether the patient was fluid responsive.

#### SV group

Intravenous lactated Ringer's solution was administered at 1 ml/kg/hr. Patients received additional 250 ml fluid challenges with intravenous colloid solution to achieve an optimal value of stroke volume. Colloid solution was administered in one or more rapid boluses to identify whether the patient was fluid responsive. A stroke volume response to fluid was defined as a sustained rise in stroke volume of at least 10% for 20 minutes or more. When a patient was identified as stroke volume responsive to fluid, further 250 ml boluses of fluid were administered until a plateau value was achieved. If stroke volume decreased, fluid challenges were repeated to establish whether the patient was fluid responsive.

#### SV & DPX group

Intravenous lactated Ringer's solution was administered at 1 ml/kg/hr. Patients received additional fluid challenges with colloid solution to achieve an optimal value of stroke volume in an identical fashion to patients in the SV group. In addition, a continuous intravenous infusion of dopexamine was administered at 0.5 μg/kg/min (Cephalon, Welwyn Garden City, UK). This infusion rate was not adjusted to achieve a specific value for cardiac output or DO_2 _index (DO_2_I) but was decreased or discontinued in patients with evidence of myocardial ischaemia or tachycardia (> 100 bpm or increase > 20% from baseline value, whichever was greater).

Only the member of the research team who delivered the intervention was aware of the study group allocation. Cardiac output data were made available to clinical staff only on specific request. The reasons for this and any subsequent changes in treatment were documented by research staff. Dummy infusions were used in patients not allocated to receive dopexamine. All other management decisions were taken by clinical staff.

### Sublingual microvascular flow

Sublingual microvascular flow was evaluated before surgery and at 0, 2, 4, 6 and 8 hours immediately after surgery using sidestream darkfield (SDF) imaging with a ×5 objective lens (Microscan, Microvision Medical, Amsterdam, Netherlands) [32]. Image acquisition and subsequent analysis was performed according to published consensus criteria [33]. SDF images were obtained from at least three sublingual areas at each time point giving a total of twelve quadrants for analysis. Vessel density was calculated by inserting a grid of three equidistant horizontal and three equidistant vertical lines over the image. Vessel density is equal to the number of vessels crossing these lines divided by their total length. Flow was then categorised as present, intermittent or absent to calculate the proportion of perfused vessels and thus the perfused vessel density. Microvascular Flow Index (MFI) was calculated after dividing each image into four equal quadrants. Quantification of flow was determined using an ordinal scale (0: no flow, 1: intermittent flow, 2: sluggish flow, 3: normal flow) for small (< 20 μm) and large (> 20 μm) vessels. MFI is the average score of all quadrants for a given category of vessel size at a given time point. Analysis of the videos was performed by two observers (AVS and SLA). The Kappa coefficient (κ) for inter-observer variability in SDF image analysis was 0.74 (95% confidence interval 0.61 to 0.81). Baseline sublingual large vessel MFI (> 20 μm) was 3.0 (3.0 to 3.0) in all groups suggesting good quality image capture unaffected by pressure artefact.

### Cutaneous microvascular flow and PtO_2_

Cutaneous red blood cell flux was measured before surgery and at 0, 4 and 8 hours after surgery at two sites on the forearm by laser Doppler flowmetry (Moorlab, Moor Instruments, Axminster, UK). Baseline red cell flux on the forearm was measured and following this, the post-occlusive hyperaemic response was examined by inflating a cuff around the upper arm to 20 mmHg above systolic pressure for three minutes and measuring the changes in red cell flux on releasing the pressure in the cuff. The difference between baseline flux and peak hyperaemia was then evaluated at each time point. Cutaneous tissue oxygen partial pressure (PtO_2_) was measured before surgery and at hour 0, 2, 4, 6 and 8 hours after surgery at two sites on the abdominal wall using a Clark electrode (TCM400, Radiometer, Copenhagen, Denmark). PtO_2 _probes warm the skin to 44°C minimising artefact due to local vasoconstriction.

### Arterial and venous blood gas analysis

Arterial and central venous blood samples were taken at hour 0, 2, 4, 6 and 8 after surgery from indwelling catheters for analysis of arterial haemoglobin saturation with oxygen, ScvO_2_, base deficit and serum lactate (ABL600, Radiometer, Copenhagen, Denmark).

### Serum inflammatory markers

Serum samples were obtained from all patients following induction of anaesthesia but prior to surgery. Further serum samples were obtained immediately following surgery, at the end of the intervention period and 24 hours after the end of surgery. These samples were centrifuged at 3,000 g for 10 minutes and stored at -80°C. Subsequent analysis of IL1 beta, IL6, IL8, and TNFα was performed using a multi-array electro-chemiluminescence technique (SECTOR Imager 2400, Mesoscale Discovery, Gaithersburg, Maryland, USA). Levels of soluble inter-cellular adhesion molecule 1 (ICAM-1) were quantified using a similar technique.

### Clinical follow-up

Clinical outcomes data for each patient were collected by a member of the research team who was unaware of study group allocation and then verified by the senior investigator who was also unaware of the study group allocation. Estimated glomerular filtration rate (eGFR) was calculated preoperatively and on day seven after surgery from serum creatinine, age, race and gender using the Modification of Diet in Renal Disease equation [34]. Patients were prospectively followed for 28 days for pre-defined in-hospital complications, including acute kidney injury within seven days [35], mortality and duration of hospital stay.

### Statistical analysis

Assuming a 5% type I error rate and an 80% type II error rate, it was calculated that 45 patients would be required in each group to detect a 10 mmHg difference in PtO_2 _between each of the intervention groups and the control group. Trends in physiological variables over time within groups were tested using one-way repeated measures analysis of variance (ANOVA) or Friedmann test. Differences in physiological variables between groups were tested using two-way repeated measures ANOVA, the t test and one-way ANOVA with *post hoc *t-test with Bonferroni correction. Categorical variables were tested with the Chi squared or Fisher's exact tests. Statistical analysis was performed using GraphPad Prism version 4.0 (GraphPad Software, San Diego, California USA). Analysis was performed on an intention-to-treat basis including all randomised patients. Significance was set at *P *< 0.05. Data are presented as mean (standard deviation) where normally distributed or median (interquartile range) where not normally distributed.

## Results

**Figure 1:**
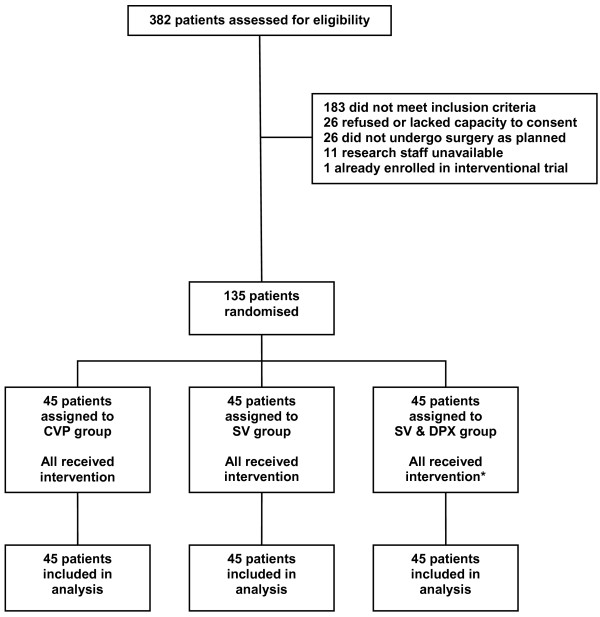
**CONSORT diagram; flow of patients through trial**. *One patient randomised to the SV & DPX group developed myocardial ischaemia during surgery (before the trial intervention commenced) and, in accordance with the protocol, did not receive dopexamine. CVP, central venous pressure; DPX, dopexamine; SV, stroke volume.

Between December 2007 and February 2009, 135 patients were recruited (Figure 1). Baseline patient characteristics are presented in Table 1. Despite the different haemodynamic treatment algorithms, patients in the three groups received similar volumes of fluid during and after surgery and there were no differences in vasopressor requirements (Table 2). The number of patients who received transfused blood during and after surgery was similar between the groups as was the volume of blood transfused (CVP group: 19 patients, 870 (580 to 1408) ml; SV group: 12 patients, 561 (398 to 580) ml; SV & DPX group: 15 patients, 580 (300 to 877) ml; *P *= 0.11). One patient randomised to the SV & DPX group developed myocardial ischaemia during surgery and, in accordance with the study protocol, did not receive dopexamine. In five patients the dose of dopexamine was reduced because of an increase in heart rate and in one patient, dopexamine was subsequently discontinued. On only one occasion, a clinician asked to view a patient's cardiac output data because of concern that poor cardiac function might have been complicated by pulmonary oedema. This information did not prompt any changes in treatment. No patients received additional inotropic therapy during the intervention period.

**Table 1:** Patient characteristics at baseline

	CVP group *n *= 45	SV group *n *= 45	SV & DPX group *n *= 45
**Age (years)**	70 (64-78)	68 (59-77)	65 (59-74)
**Male (%)**	30 (67%)	31 (69%)	28 (62%)
**ASA score**	2 (2-3)	2 (2-3)	2 (2-3)
**Upper gastrointestinal surgery (%)**	18 (40%)	18 (40%)	18 (40%)
**Pancreatic surgery involving gut (%)**	18 (40%)	18 (40%)	18 (40%)
**Lower gastrointestinal surgery (%)**	9 (20%)	9 (20%)	9 (20%)

Data presented as median (IQR) or absolute values (%). ASA, American society of anesthesiologists; CVP, central venous pressure; DPX, dopexamine; SV, stroke volume.

**Table 2:** Volume of intravenous fluid administered and use of vasopressor therapy in the three groups

	CVP group *n *= 45	SV group *n *= 45	SV & DPX group *n *= 45	*P*
**Intra-operative period**				
Intravenous crystalloid during surgery (ml)	3595 (1354)	4057 (1495)	4159 (1393)	0.15
Intravenous colloid during surgery (ml)	756 (815)	835 (688)	709 (559)	0.69
**Intervention period**				
Intravenous crystalloid during study period (ml)	639 (281)	652 (237)	626 (250)	0.98
Intravenous colloid during study period (ml)	1104 (553)	1227 (555)	1307 (549)	0.22
Patients receiving vasopressor therapy (%)	7 (16%)	8 (18%)	5 (11%)	0.82

Data presented as mean (standard deviation) or absolute values (%). CVP, central venous pressure; DPX, dopexamine; SV, stroke volume.

**Figure 2:**
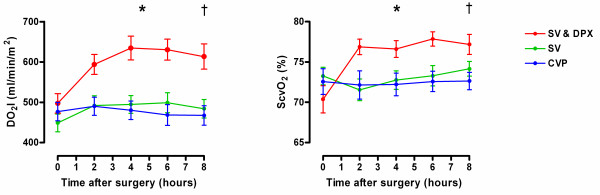
**Changes in (a) oxygen delivery index and (b) central venous oxygen saturation following surgery in the three treatment groups**. *Significant difference between groups over time for oxygen delivery index (DO_2_I) and central venous oxygen saturation (ScvO_2_; *P *< 0.0001; two-way repeated measures analysis of variance). Significant increase in DO_2_I over time: SV group *P *= 0.003; SV & DPX group *P *< 0.0001. Significant increase in ScvO_2 _over time: SV & DPX group *P *< 0.0001; no change in the SV group (*P *= 0.22) or CVP group (*P *= 0.98). †At hour eight, there was a significant difference in DO_2_I between the CVP and SV & DPX groups (*P *< 0.001) but no difference between the SV and CVP groups (*P *> 0.05). At hour eight, there was a significant difference in ScvO_2 _between the CVP and SV & DPX groups (*P *< 0.05) but no difference between the SV and CVP groups (*P *> 0.05). CVP, central venous pressure; DPX, dopexamine; SV, stroke volume.

**Figure 3:**
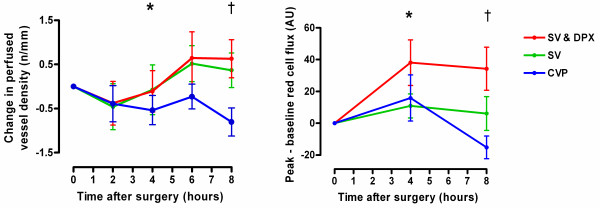
**Changes in (a) sublingual perfused vessel density and (b) peak-baseline cutaneous red cell flux following three minutes of vascular occlusion from hour 0 following surgery in the three treatment groups**. *Significant difference between groups over time for sublingual vessel density (*P *< 0.05) and cutaneous hyperaemic response (*P *< 0.01) (two-way repeated measures analysis of variance). Significant increase in perfused sublingual vessel density over time in the SV & DPX group (*P *= 0.046), no change in the SV group (*P *= 0.58) and a decrease in the CVP group (*P *= 0.005). Significant increase in cutaneous hyperaemic response over time in the SV & DPX group (*P *= 0.003), no change in the SV group (*P *= 0.58) and a decrease in the CVP group (*P *= 0.03). †At hour eight, there was a significant difference in perfused sublingual vessel density between the SV & DPX and CVP groups (*P *< 0.05) but not between the SV and CVP groups (*P *> 0.05). At hour eight, there was a significant difference in cutaneous hyperaemic response between the SV & DPX and CVP groups (*P *< 0.001) but not between the SV and CVP groups (*P *> 0.05). CVP, central venous pressure; DPX, dopexamine; SV, stroke volume.

**Figure 4:**
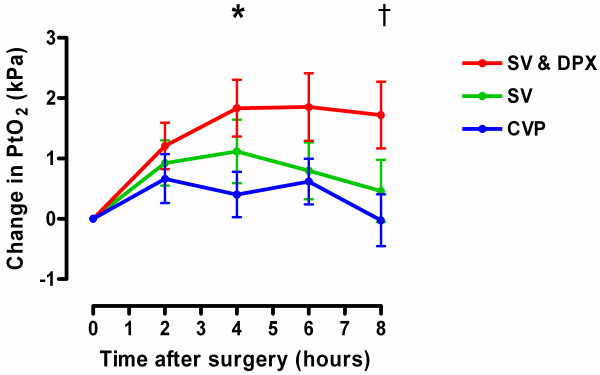
**Changes in tissue oxygenation following surgery in the three treatment groups**. *Significant difference between groups over time (*P *= 0.0005; two-way repeated measures analysis of variance). Significant increase in tissue oxygenation (PtO_2_) over time in the SV & DPX group (*P *= 0.0003), no change in the SV (*P *= 0.14) or CVP groups (*P *= 0.20). †At hour eight, there was a significant difference in PtO_2 _between the SV & DPX and CVP groups (*P *< 0.005) but not between the SV and CVP groups (*P *> 0.05). CVP, central venous pressure; DPX, dopexamine; SV, stroke volume.

Stroke volume-guided fluid therapy with dopexamine infusion was associated with significant increases in heart rate, cardiac index, DO_2 _and ScvO_2_. Stroke volume-guided fluid therapy alone was associated with much smaller increases in cardiac index and DO_2 _and no change in heart rate or ScvO_2 _(Figure 2 and Table 3). In all three groups, microvascular flow was impaired at baseline (Table 4). In the SV & DPX group, sublingual microvascular flow significantly improved during the eight-hour study period (Figure 3 and Table 4). Sublingual microvascular flow remained constant in the SV group but deteriorated in the control group (Figure 3). Similarly, there was a significant improvement in the cutaneous hyperaemic response in the SV & DPX group, whereas this variable remained unchanged in the SV group and deteriorated in the control group (Figure 3). In all three groups, cutaneous PtO_2 _initially increased after surgery. This improvement was sustained in the SV & DPX group but decreased towards baseline in the CVP and SV groups (Figure 4).

**Table 3:** Cardiovascular physiology for the three treatment groups during eight hour study period

	Group	Hour 0	Hour 2	Hour 4	Hour 6	Hour 8
**Heart rate** **(bpm)**	CVP	74 (13)	76 (14)	76 (14)	76 (15)	76 (15)
	SV	77 (19)	76 (17)	80 (19)	79 (17)	78 (17)
	§ SV & DPX	77 (11)	86 (12)	91 (12)	93 (13)	92 (12)
**Mean arterial pressure (mmHg)**	CVP	80 (22)	79 (20)	79 (15)	79 (15)	77 (14)
	SV	76 (15)	81 (15)	83 (14)	79 (14)	77 (14)
	† SV & DPX	80 (18)	83 (17)	84 (13)	77 (13)	74 (12)
**Central venous pressure (mmHg)**	‡ CVP	6 (5)	7 (5)	7 (5)	8 (5)	8 (5)
	* SV	4 (5)	6(4)	6 (5)	7 (4)	7 (5)
	† SV & DPX	5 (4)	7 (4)	7 (4)	8 (6)	8 (5)
**Cardiac index** **(l/min/m^2^)**	CVP	3.5 (1.1)	3.5 (0.9)	3.5 (0.9)	3.5 (0.9)	3.4 (0.9)
	‡ SV	3.2 (0.9)	3.5 (0.9)	3.7 (1.0)	3.7 (1.0)	3.6 (1.0)
	§ SV & DPX	3.3 (0.8)	4.0 (0.9)	4.3 (1.0)	4.3 (0.9)	4.4 (1.1)
**Oxygen delivery index** **(ml/min/m^2^)**	CVP	477 (146)	490 (144)	480 (152)	468 (168)	467 (159)
	† SV	449 (145)	492 (160)	495 (147)	499 (165)	484 (150)
	§ SV & DPX	498 (157)	594 (167)	635 (198)	631 (174)	614 (209)
**Stroke volume** **(ml)**	CVP	80 (23)	86 (25)	84 (24)	82 (21)	81 (21)
	‡ SV	78 (23)	85 (22)	84 (22)	85 (22)	83 (22)
	‡ SV & DPX	80 (23)	88 (24)	90 (24)	89 (23)	88 (26)
**Serum lactate** **(mmol/l)**	† CVP	1.4 (1.0-2.1)	1.1 (0.9-1.6)	1.1 (0.9-1.8)	1.2 (0.9-1.8)	1.2 (0.9-1.8)
	* SV	1.4 (0.9-2.7)	1.3 (0.9-2.2)	1.3 (0.8-2.4)	1.2 (0.8-1.9)	1.2 (0.8-1.8)
	SV & DPX	1.9 (1.3-2.8)	1.7 (1.0-2.4)	1.9 (1.0-2.9)	1.9 (1.0-3.1)	1.7 (1.1-2.4)
**Base deficit** **(mmol/l)**	CVP	-1.9 (2.6)	-2.2 (2.7)	-1.7 (2.8)	-1.7 (2.9)	-1.6 (2.6)
	* SV	-2.2 (2.4)	-2.1 (2.8)	-1.6 (3.1)	-1.0 (2.2)	-1.0 (2.3)
	‡ SV & DPX	-2.2 (2.1)	-2.3 (2.4)	-2.2 (2.4)	-1.9 (2.3)	-1.4 (2.4)

Data presented as mean (standard deviation) or median (interquartile range). Significant changes over time signified by † (*P *< 0.05), ‡ (*P *< 0.01), * (*P *< 0.001) and § (*P *< 0.0001). CVP, central venous pressure; DPX, dopexamine; SV, stroke volume.

**Table 4:** Sublingual microvascular flow for small vessels (< 20 μm) during eight hour study period

		Hour 0	Hour 2	Hour 4	Hour 6	Hour 8
**Microvascular Flow Index**	CVP	2.5 (0.3)	2.5 (0.7)	2.6 (0.4)	2.6 (0.4)	2.5 (0.5)
	SV	2.5 (0.4)	2.5 (0.5)	2.6 (0.4)	2.7 (0.3)	2.6 (0.4)
	† SV & DPX	2.5 (0.4)	2.4 (0.5)	2.5 (0.4)	2.7 (0.3)	2.5 (0.4)
**Perfused vessel density** **(mm^-1^)**	‡ CVP	6.1 (2.4)	6.1 (1.7)	5.8 (2.0)	5.8 (1.9)	5.3 (1.8)
	SV	5.8 (2.5)	5.7 (2.6)	5.7 (1.9)	5.7 (1.9)	6.2 (3.0)
	† SV & DPX	5.8 (2.4)	5.5 (2.4)	5.9 (2.8)	6.2 (1.8)	6.3 (3.0)
**Proportion of perfused vessels**	CVP	0.83 (0.14)	0.83 (0.12)	0.81 (0.14)	0.82 (0.18)	0.81 (0.18)
	SV	0.80 (0.15)	0.80 (0.21)	0.82 (0.17)	0.84 (0.13)	0.80 (0.19)
	SV & DPX	0.81 (0.16)	0.77 (0.14)	0.81 (0.15)	0.85 (0.12)	0.87 (0.17)
**Heterogeneity index**	CVP	0.39 (0.23-0.51)	0.23 (0.12-0.41)	0.25 (0.17-0.48)	0.28 (0.16-0.38)	0.25 (0.10-0.54)
	SV	0.23 (0.10-0.43)	0.20 (0.07-0.31)	0.22 (0.04-0.41)	0.19 (0.06-0.31)	0.22 (0.08-0.46)
	† SV & DPX	0.27 (0.18-0.40)	0.20 (0.14-0.44)	0.18 (0-0.27)	0.13 (0.08-0.27)	0.17 (0-0.38)

Data presented as mean (standard deviation) or median (interquartile range). Significant changes over time signified by † (*P *< 0.05), ‡ (*P *< 0.01). CVP, central venous pressure; DPX, dopexamine; SV, stroke volume.

**Figure 5:**
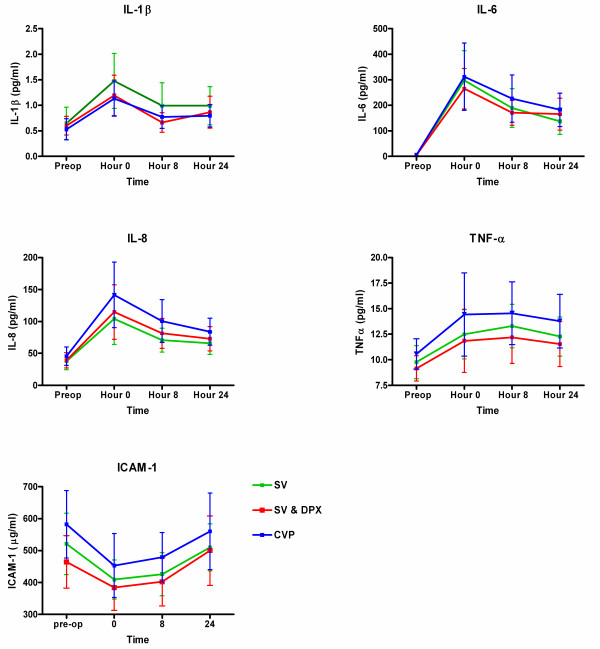
**Changes in (a) serum IL-1β, (b) IL-6, (c) IL-8, (d) TNFα and (e) soluble inter-cellular adhesion molecule 1 between the three treatment groups**. Data presented as mean (standard error). There were no significant differences between the groups. CVP, central venous pressure; DPX, dopexamine; ICAM-1, inter-cellular adhesion molecule 1; SV, stroke volume.

There were no significant differences in overall complication rates, critical care free days or duration of hospital stay, although the pattern of mortality was consistent with a beneficial effect of stroke volume-guided haemodynamic therapy (Table 5). During the first seven days after surgery, eGFR increased significantly in the SV & DPX group but not in the SV or the CVP group (SV & DPX group 21 [20] ml/min, *P *= 0.001; SV group 10 [33] ml/min, *P *= 0.09; CVP group 2 [35] ml/min; *P *= 0.73). Consequently, a *post hoc *analysis of the predefined renal outcome was performed. Fewer patients developed acute kidney injury in the pooled SV and SV & DPX groups within seven days of surgery (*P *= 0.03; Table 5). Despite improvements in tissue microvascular flow and oxygenation in the SV and SV & DPX groups, there were no differences between the groups in terms of the serum inflammatory markers IL-1β, IL-6, IL-8, TNFα and ICAM-1 within 24 hours of surgery (Figure 5).

**Table 5:** Clinical outcomes in the three intervention groups

	CVP group *n *= 45	SV group *n *= 45	SV & DPX group *n *= 45	*P*
Complications (number of patients, %)	30 (67%)	26 (58%)	31 (69%)	0.51
Cardiac complications (number of patients, %)	4 (9%)	3 (7%)	3 (7%)	0.90
Infectious complications (number of patients, %)	29 (64%)	24 (53%)	28 (62%)	0.52
Other complications (number of patients, %)	10 (22%)	14 (31%)	12 (27%)	0.63
Acute kidney injury within 7 days of surgery	10 (22%)	3 (7%)	4 (9%)	0.055*
Critical care free days within 28 days of surgery	24 (21-26)	24 (21-26)	26 (21-27)	0.45
Duration of hospital stay (days)	15 (10-26)	14 (11-26)	16 (11-28)	0.73
Hospital mortality (%)	6 (13%)	5 (11%)	4 (9%)	0.45

Data presented as median (interquartile range) or absolute values (%). Note: A number of patients developed more than one complication. Acute kidney injury at seven days not included in 28 day complication outcome.

*Significant difference in incidence of acute kidney injury between pooled SV and SV & DPX groups and the CVP group (*P *= 0.03, *post hoc *analysis). CVP, central venous pressure; DPX, dopexamine; SV, stroke volume.

## Discussion

This is the first study to substantiate the theory that cardiac output-guided haemodynamic therapy can improve tissue perfusion and oxygenation. Our principal finding is that a treatment algorithm incorporating stroke volume-guided fluid therapy and a low-dose dopexamine infusion increased global DO_2 _and ScvO_2 _in association with significant improvements in sublingual and cutaneous microvascular flow and cutaneous tissue oxygenation. Stroke volume-guided fluid therapy alone was associated with more modest improvements in global haemodynamics and microvascular flow. There were, however, no differences in circulating markers of the inflammatory response to surgery between treatment groups.

This randomised controlled trial used physiological end-points and was not designed to identify differences in clinical outcomes although a *post hoc *analysis did identify a possible improvement in renal outcomes (eGFR and incidence of acute kidney injury) associated with stroke volume-guided therapy. This finding is consistent with a recent meta-analysis suggesting that haemodynamic optimisation protects renal function in surgical patients [31]. There was no reduction in overall complication rates in the intervention groups and the small difference in hospital mortality, although consistent with improved outcome was not significant. To achieve 80% power to detect a 25% reduction in the relative risk of complications would require a minimum of 150 patients in each of the three treatment groups.

In common with all trials of complex interventions, it was not possible to fully blind clinical staff to study group allocation. We did, however, conceal study group allocation from all investigators apart from the member of the research team delivering the intervention. This included concealment of cardiac output data and the use of dummy infusions. All complications, including acute kidney injury, were assessed according to prospectively defined criteria and verified by the principal investigator who was unaware of study group allocation. Lastly our stratified randomisation procedure ensured that the three groups were comparable.

The importance of using cardiac output-derived data to guide a carefully prescribed and consistently applied clinical intervention is illustrated by the findings of a previous multi-centre randomised trial in which perioperative pulmonary artery catheterisation, in the absence of improved haemodynamics, failed to influence outcome [36]. In the study reported here, three clinically relevant treatment algorithms were strictly implemented by members of the research team throughout the eight-hour intervention period. Perhaps as a consequence, unlike most previous studies, the total volumes of intravenous fluid administered were similar between the groups [12-18]. This suggests a high standard of care for all patients that may have limited the apparent treatment effect of stroke volume-guided fluid therapy. Interestingly, the findings of one previous trial suggest that, even where median fluid administration is similar between groups, cardiac output-guided fluid therapy may be associated with improved clinical outcomes [37].

The relation between derangements in cardiac output-related variables and complications following major surgery is well described [9-11]. The findings of some, but not all clinical trials and a number of meta-analyses suggest that cardiac output-guided haemodynamic therapy can improve post-operative outcomes [12-18,30,31]. It has long been assumed that the potential benefits of 'flow guided' peri-operative haemodynamic therapy relate to improved tissue perfusion and oxygenation. A number of studies have highlighted the significance of impaired tissue microvascular flow in the pathogenesis of post-operative complications [21-24]. In this context, it is interesting to note that the use of high concentrations of inspired oxygen did not affect the incidence of post-operative wound infection or pneumonia in a recent large clinical trial [38]. In the current study, the use of a fixed low-dose inotrope infusion coupled with stroke volume-guided fluid therapy resulted in increases in heart rate and, to a lesser extent, stroke volume which in turn increased DO_2 _and ScvO_2 _to values previously associated with improved clinical outcomes [9-11]. We show for the first time that such increases in global haemodynamics are associated with improvements in tissue microvascular flow and oxygenation, thus validating the study's hypothesis. Although stroke volume-guided intravenous colloid therapy led to much smaller increases in cardiac index and DO_2_, with no change in heart rate or ScvO_2_, microvascular flow was better maintained than in the CVP-guided therapy group. The incremental effects of low dose dopexamine on both microvascular flow and tissue oxygenation are likely to relate to the β_2_-adrenoceptor-mediated inotropic and vasodilator actions of this agent. It is therefore possible that changes in microvascular flow relate to direct effects on the microcirculation as well as global cardiac output.

Interestingly, in a recent randomised trial, low-dose nitroglycerin had no effect on sublingual microvascular flow in resuscitated patients with severe sepsis [39]. These contrasting findings may reflect differences in the nature and timing of the intervention as well as the patient population and smaller sample size. In contrast, the use of vasopressor and inotropic agents has been shown to improve both tissue microvascular flow and oxygenation in patients with severe sepsis [28,29], although these effects were not demonstrated in all such investigations [40,41]. While, these studies do suggest potential effects of vasoactive drugs on microvascular flow, the current study is the first to investigate the effects of the use of cardiac output-based end-points on tissue microvascular flow and oxygenation.

The simultaneous use of three different modalities to assess different aspects of tissue microvascular function was an important strength of this investigation. SDF imaging is a non-invasive technique that provides a real-time video image of the intact microcirculation. However, this technique is limited by semi-quantitative analysis and the fact that it can only be used to image the microcirculation under mucosal surfaces. Laser Doppler flowmetry is a technique based on the Doppler shift of reflected laser light from moving red blood cells. This method cannot distinguish the size or type of microvessel, direction of flow or heterogeneity of flow, all of which may be important in critically ill or high-risk surgical patients. These limitations can be addressed through the measurement of post-occlusion reactive hyperaemia, which provides a reproducible assessment of endothelium-dependant microvascular response [42]. The cutaneous Clarke electrode measures the local partial pressure of oxygen by a polarographic method. If tissue perfusion decreases while partial pressure of oxygen (PaO_2_) remains constant, cutaneous PtO_2 _will decrease thus linking peripheral perfusion and tissue oxygenation [43]. The consistent patterns of change identified with each of the three modalities is therefore of particular importance. However, these methods have been used to assess quite different aspects of microvascular function and cannot be directly compared. We have presented the changes in microvascular flow in terms of change from the baseline values. While differences are less apparent on analysis of absolute values, the consistency of the changes we observed between three distinct measures of tissue perfusion strongly suggests that these findings are robust.

## Conclusions

A treatment algorithm incorporating stroke volume-guided fluid therapy plus low-dose dopexamine infusion was associated with significant improvements in microvascular flow and tissue oxygenation but no change in the inflammatory response to surgery. These physiological changes may explain the beneficial effects of cardiac output-guided haemodynamic therapy demonstrated in previous clinical trials. Our findings strongly support the need for large multi-centre trials to evaluate the clinical effectiveness of cardiac output-guided haemodynamic therapy. Several such trials are now under way in patients with severe sepsis, those undergoing major surgery and in potential organ donors.

## Key messages

• Peri-operative haemodynamic therapies guided by cardiac output monitoring have been associated with improved clinical outcomes in small clinical trials.

• The mechanism of therapeutic benefit is believed to relate to improved tissue perfusion and oxygen delivery but this theory has not previously been tested.

• In this study, stroke volume-guided fluid therapy and low-dose dopexamine infusion was associated with improvements in tissue microvascular flow and oxygenation but clinical outcomes were similar between groups.

• These findings may explain the improved clinical outcomes reported in previous studies. Large randomised trials are now required to confirm the clinical benefits of this treatment approach.

## Abbreviations

ANOVA: analysis of variance; CVP: central venous pressure; DO_2_: oxygen delivery; DO_2_I: oxygen delivery index; DPX: dopexamine; eGFR: estimated glomerular filtration rate; ICAM-1: inter-cellular adhesion molecule 1; IL: interleukin; MFI: microvascular flow index; PaCO_2_: partial pressure of arterial carbon dioxide; PaO_2_: partial pressure of arterial oxygen; PtO_2_: tissue oxygen partial pressure; SaO_2_: arterial haemoglobin saturation with oxygen; ScvO_2_: central venous haemoglobin saturation with oxygen; SpO_2_: arterial haemoglobin saturation; SV: stroke volume; SvO_2_: mixed venous haemoglobin saturation with oxygen; SDF: sidestream darkfield imaging; TNF: tumour necrosis factor.

## Competing interests

RP has received a research grant and equipment loans from LiDCO Ltd and honoraria from BBraun, Edwards Lifesciences, Covidien and Pulsion Medical Systems. SJ, CH and RP are named inventors on a lapsed patent application relating to the peri-operative use of dopexamine.

## Authors' contributions

RP formulated the hypothesis and developed the protocol with CH and DW. The investigation was performed by SJ, AVS and SLA, at The Royal London Hospital, UK. SJ, RP, DW and CH assisted in the data analysis. The manuscript was drafted by SJ and RP. All authors read and approved the final version.
